# Effects of dietary energy and essential amino acid reduction on injuries, plumage damage, and plumage pigmentation of female slow- (Auburn) and fast-growing (B.U.T. 6) Turkey strains under organic feeding conditions

**DOI:** 10.1016/j.psj.2025.105797

**Published:** 2025-09-04

**Authors:** A.I. Kirn, P. Hofmann, P.A. Weindl, C. Lambertz, G. Bellof, R. Schreiter

**Affiliations:** a Weihenstephan-Triesdorf University of Applied Sciences, Am Staudengarten 1, Freising 85354, Germany; b Bavarian State Research Center for Agriculture, Kitzingen, Germany; c Research Institute of Organic Agriculture, Witzenhausen, Germany; d Martin Luther University Halle-Wittenberg, Halle, Germany

**Keywords:** Organic female turkey feeding, Dietary energy and essential amino acid reduction, Injurious pecking, Plumage damage, Plumage pigmentation

## Abstract

The objective of this study was to investigate the effects of reduced dietary AME_N_ and essential amino acid (**EAA**) concentrations on injuries, plumage damage, and plumage pigmentation in two female turkey strains. 216 day-old, non-beak-trimmed Auburn and B.U.T. 6 turkeys were assigned to three feeding groups and studied over four 4-week phases. Diets were formulated per phase with a 10 % isoenergetic AME_N_ reduction compared to breeder recommendations. Feeding groups differed in EAA levels, with methionine and lysine relative to breeder recommendations as follows: F1 (80/90/90/90 %), F2 (80/80/90/90 %), and F3 (70/80/90/90 %). Injuries, plumage damage, and plumage pigmentation were evaluated using a scoring system and a longitudinal visual assessment. Data, including growth performance, were collected during rearing (weeks 4 and 8) and fattening (weeks 12 and 16), at the end of each phase. B.U.T. 6 turkeys showed a higher prevalence of plumage damage, feather structure alterations, and skin injuries compared to Auburn turkeys (*P* < 0.001). A reduction in EAA by up to 30 % (F3) resulted in increased skin injuries, plumage damage, alterations in wing feather structure, and depigmentation (*P* ≤ 0.010). An interaction between genotype and feeding was observed *(P* = 0.048), with the increase in animals showing altered feather structure in F2 and F3 compared to F1 being more pronounced in Auburn than in B.U.T. 6. While pecking injuries and plumage damage increased with age *(P* ≤ 0.019), feather structure alterations and depigmentation decreased with age *(P* < 0.001) and higher EAA supply levels, suggesting these alterations are reversible. This study highlights the crucial role of dietary EAA in maintaining plumage and skin condition in female turkeys. Reducing EAA levels by up to 30 % resulted in more pronounced effects, with wing feathers proving to be a useful longitudinal tool to assess amino acid status in turkeys. However, reducing EAA levels by 20 % during rearing maintained final growth performance without adverse effects on injuries, plumage, or pigmentation, but EAA reduction should be carefully monitored in practice.

## Introduction

Injuries and feather pecking are major animal welfare issues in turkey farming (Haug et al., 2023). These behavioral disorders are influenced by endogenous factors like genotype (**G**), age, and sex, as well as exogenous factors, including environment and nutrition (Kulke et al., 2016). While the relationship between feed formulation and feather pecking is well-studied in laying hens (Jong et al., 2013; Hartcher et al., 2016; Mens et al., 2020), it is also relevant for turkeys (Dalton et al., 2013). However, the effects of dietary amino acid (**AA**) imbalances in turkeys remain insufficiently studied. Nutritional imbalances, such as AA deficiencies, can directly influence physiological mechanisms that trigger feather pecking in poultry (Mens et al., 2020; Nikolov and Kanakov, 2020). Implementing a nutritional strategy optimized with AA and fiber during the sensitive rearing phase has been proposed to mitigate the development of feather pecking (Dalton et al., 2013; Mens et al., 2020; Nikolov and Kanakov, 2020).

However, in organic poultry systems, AA optimization is constrained by EU Regulation 2018/848, which prohibits the use of free AA in poultry diets (Guarino Amato and Castellini, 2022). This limitation often leads to unbalanced feed formulations with elevated crude protein contents to compensate for AA deficiencies, increasing environmental nitrogen emissions (Ferket et al., 2002; Blair, 2018). Thus, a balanced feeding strategy that optimizes AA and energy content while reducing nitrogen output is crucial for sustainable organic poultry production (Lindberg, 2023). One approach to enhancing nitrogen utilization efficiency while maintaining growth performance in slow-growing (Auburn) and fast-growing (B.U.T. 6) male turkeys involves a gradual adjustment of dietary essential amino acid (**EAA**) levels, focusing on methionine and lysine (Göppel et al., 2022). This includes reducing EAA by up to 20 % compared to the breeder's recommendations (Aviagen, 2015) during rearing, followed by a gradual increase during fattening to 90-100 % of the breeder’s recommendations. Combined with a consistent 10 % reduction in AME_N_ throughout rearing and fattening, this approach leverages compensatory feed intake and growth to sustain final growth performance. The performance of these turkeys did not differ from those continuously fed with 100 % EAA according to breeder’s recommendations (Göppel et al., 2022). Recent findings suggest that even stronger EAA reductions, by up to 30 % during rearing, do not negatively affect final body weight (**BW**) and valuable cuts in male turkeys (Kirn et al., 2024a).

In addition to the potential link between dietary EAA reduction and the prevalence of injuries and plumage damage, previous research suggest that reduced levels of EAA may influence feather structure and pigmentation (Deschutter and Leeson, 1986; Leeson and Summers, 2008; Kirn et al., 2024b). This is particularly relevant as changes in feathers can reflect the nutritional status of poultry (Leishman et al., 2020; Mróz et al., 2022), providing an early indicator of potential deficiencies. However, these effects under reduced EAA and AME_N_ conditions have not yet been systematically studied in turkeys. To address this gap, this study investigates the effects of reducing AME_N_ by 10 % and EAA levels by up to 30 % compared to breeder’s recommendations on injuries, plumage damage, feather structure, and pigmentation in female Auburn and B.U.T. 6 turkeys. A comprehensive longitudinal approach was established to provide deeper insights into how these dietary adjustments influence feather structure and pigmentation across different turkey G. The study's hypotheses were as follows: 1) A significant reduction in EAA will increase the prevalence of changes in feather structure and depigmentation. 2) G will differ in their response to EAA feeding (**F**) in terms of plumage condition and injuries.

## Materials and methods

The data presented herein were collected as part of a larger research project, which investigates the impact of various G, F strategies, and housing systems on behavior, welfare, growth performance, and slaughter performance of turkeys.

### Ethical statement

The study, including all procedures, was conducted in accordance with the provisions of the German Animal Welfare Act and the European Union Guidelines (2010/63/EU) and was approved by the Animal Welfare Officer and Committee of the Weihenstephan-Triesdorf University of Applied Science (Permit-Number: HSWT-2022-1).

### Birds and housing

The feeding trial was conducted from August to December 2023 at the Bavarian State Estate Kitzingen, Experimental and Educational Center for Poultry Husbandry (Kitzingen, Germany). A total of 216 day-old non-beak-trimmed female hatchlings of two turkey strains (Ayrshire Auburn, B.U.T. 6; Aviagen Turkeys Ltd, Chowley Five, Chowley Oak Business Park, Tattenhall, Cheshire, CH3 9GA, United Kingdom) were purchased from the hatchery Moorgut Kartzfehn Turkey Breeder GmbH (Bösel, Germany). The 2 × 3 experimental setup included two G and three F strategies, with four replicates per treatment. Day-old poults were randomly allocated based on individual BW to one of three dietary treatments, with 9 birds per pen, resulting in a total of 24 pens (12 pens per G). Allocation ensured that each pen within a G had a similar average BW. The trial included four F phases of four weeks each (P1-P4), divided into rearing (P1-P2) and fattening (P3-P4) periods (Table 1).

**Table 1:** Experimental design.

Feeding phase				Essential amino acid levels (%)^1^ of the feeding groups (F)		
Stage	Phase	Week	Days	F1	F2	F3
Rearing	P1	1-4	1-28	80	80	70
	P2	5-8	29-56	90	80	80
Fattening	P3	9-12	57-84	90	90	90
	P4	13-16	85-112	90	90	90

1 Essential amino acid levels (focus: lysine and methionine) used in the experimental diets compared to Aviagen (2015) recommendations for a low energy density feeding program, based on the ratio of lysine and methionine to MJ AME_N_. These calculated ratios varied in the feed mixtures used during rearing due to adjustments in lysine and methionine levels, while maintaining isoenergetic AME_N_ (see Table 2).

The turkeys were housed indoors (10 m²/pen, 1.11 m²/animal) under controlled conditions. They had no access to free-range areas or roughage, which could have contributed to their AA intake (Göppel et al., 2022; Thesing et al., 2023). This exclusion was crucial to avoid confounding interpretation of the effects of reducing dietary EAA by up to 30 % on skin and plumage condition. During rearing, the birds were kept on wood shavings (Premiumspan Profi, Hobelspanverarbeitung GmbH, Dittersdorf, Germany), and during fattening on SoftCell bedding (Desintec SoftCell, AGRAVIS Raiffeisen AG, Münster, Germany). Feed and water were provided *ad libitum* via height-adjustable drinkers (Plasson MK 2 drinker; Firma Hans Gaab, Walter Gaab, Wieseth, Germany) and feeders (capacity: 20 kg; Siepmann GmbH, Herdecke, Germany). Additional feed accessibility during the first week was ensured using feeding plates (4 cm rim height, 40 cm diameter; Siepmann GmbH, Herdecke, Germany) and egg humps (20-egg carton; Klose & Debus GbR, Ruppichteroth, Germany). The light and temperature program followed Kartzfehn´s (2021) recommendations with a light intensity of 20 lux. Grit was provided once weekly (120 g per dose/pen, quartz sand, Casafino, BayWa AG München, Germany) and pecking stones (PICKME®STARTER, Witteler GmbH & Co KG, Anröchte, Germany) were available from week two onwards. Immunization protocols included vaccinations against Newcastle disease (weeks 3, 8; Nobilis ND Clone 30, Intervet International b.v., AN Boxmeer, Netherlands), hemorrhagic enteritis (week 4; Dindoral SPF, Merial Laboratoire Porte des Alpes, France), and turkey rhinotracheitis (weeks 1, 7, 11: Terivac, Merial SAS, Lyon, France; weeks 2, 6, 10: Poulvac TRT, Zoetis Deutschland GmbH, Berlin, Germany).

### Experimental diets

The experimental diets were formulated to align with EU Organic Regulation 2018/848 and were based on a low-energy-density F program of the breeder (Aviagen, 2015). In comparison to the breeders’ recommendations (Aviagen, 2015), the AME_N_ and EAA levels were reduced. Following previous research (Göppel et al., 2022), this study implemented EAA reductions, focusing on methionine and lysine, of 10 % to 30 % during rearing and 10 % during fattening (Table 1). Other AAs were also reduced proportionally but remained above limiting thresholds. The EAA concentrations were calculated using lysine and methionine to AME_N_ ratios. The control F strategy (**F1)** was based on the recommended F strategy by Göppel et al., (2022) with the following EAA levels: 80/90/90/100 % in F phase 1, 2, 3 and 4, respectively. To investigate the effects of further reducing EAA by up to 30 %, F strategies **F2** and **F3** were formulated. In P1, both F1 and F2 had an EAA level of 80 %, but F1 contained conventional pea protein concentrate, whereas F2 excluded it. Compared to the F strategy of Göppel et al., (2022), EAA levels in P4 were reduced from 100 % to 90 % in this study. The composition of the experimental diets is shown in Table 2. All diets were formulated to be isoenergetic within each F phase, with a 10 % reduction in AME_N_ compared to Aviagen (2015) to simulate commercial organic F conditions (Göppel et al., 2022). By maintaining an isoenergetic AME_N_ level while gradually reducing EAA by 10 % to 30 % during rearing, the calculated AME_N_-to-EAA ratio of the experimental diets was deliberately varied across the first two F phases (Table 2).

**Table 2:** Composition (% of original substance), calculated AME_N_ (MJ AME_N_/kg), and the lysine and methionine to AME_N_ ratios (g/MJ) of complete feed mixtures used during rearing (phases 1 and 2) and fattening (phases 3 and 4).

Feed components	Phase 1			Phase 2		Phase 3	Phase 4
	80 %^1^	80 %	70 %	90 %	80 %	90 %	90 %
Soybean cake	12.5	27.5	12.0	14.5	13.0	14.5	9.0
Sunflower cake (45 % CP)	23.0	21.5	20.0	21.0	16.0	-	-
Sunflower cake (38 % CP)	-	-	-	11.0	9.0	22.0	7.5
Sunflower cake (30 % CP)	-	-	-	-	-	-	10.0
Pea protein concentrate	6.0	-	4.50	-	-	-	-
Peas	-	-	-	12.5	11.5	8.0	6.0
Rapeseed cake	7.0	6.5	5.0	8.0	7.0	8.5	7.0
Corn	14.2	9.6	17.6	10.5	16.5	9.1	20.0
Wheat	13.0	10.0	17.0	10.0	15.0	10.0	10.0
Triticale	-	-	-	-	-	10.0	15.0
Proso millet	-	-	-	-	-	10.0	8.5
Oat	8.0	8.0	8.0	5.0	5.0	-	-
Wheat bran	5.0	5.0	5.0	-	-	-	-
Molasses	-	-	-	2.5	2.5	2.5	2.5
Alfalfa meal	5.0	5.0	5.0	-	-	-	-
Soybean oil	1.6	2.3	1.1	1.3	0.6	2.2	1.9
Premix^2^	1.3	1.3	1.3	1.1	1.1	0.9	0.9
Calcium carbonate	1.3	1.2	1.2	1.3	1.2	1.1	1.0
Monocalcium phosphate	1.9	1.9	2.1	1.1	1.4	1.0	0.5
Sodium chloride	0.2	0.2	0.2	0.2	0.2	0.2	0.2
EcoVit R^3^	0.05	0.05	0.05	0.03	0.03	0.03	0.01
*Calculated*							
AME_N_^4^	11.5			11.9		12.3	12.4
Lysine/AME_N_^5^	1.17	1.17	1.03	1.08	0.96	0.88	0.68
Methionine/AME_N_^5^	0.41	0.41	0.37	0.39	0.34	0.32	0.27

1 Essential amino acid levels (EAA, focus: lysine and methionine) used in the experimental diets compared to Aviagen (2015) recommendations for a low energy density feeding program, based on the ratio of lysine and methionine to MJ AME_N_.

2 Premix ingredients per kg premix: Vit. A 800,000 IU; Vit. D 350,000 IU; Vit. E 5,000 mg; Vit. K3 200 mg; Vit. B1 200 mg; Vit. B6 400 mg; Vit. B12 2,000 µg; Niacin 6,500 mg; Pantothenic acid 1,600 mg; Folate 200 mg; Biotin 20,000 µg; Choline chloride 60,000 mg; Iron 4,000 mg; Copper 600 mg; Zinc 5,000 mg; Manganese 6,000 mg; Iodine 100 mg; Selenium 20 mg.

3 Eco Vit R®: organic certified fermentation product from ashbya gossypii with a riboflavin content of 6,740 mg/kg (Agrano GmbH & Co. KG, Riegel am Kaiserstuhl, Germany).

4 Calculated according to WPSA (1984).

5 The ratios represent the calculated relationship between AME_N_ and lysine and methionine, deliberately varied in Phases 1 and 2 by varying EAA levels while maintaining isoenergetic AME_N_-levels.

All diets were pelleted (3 mm), coccidiostat- and enzyme-free, and produced either at the Bavarian State Research Center (P1 diets, Poing-Grub, Germany) or Meika-Biofutter GmbH (P2 onwards, Grossaitingen, Germany). Nutrient contents were analyzed following European Commission (2009) Regulation No. 152/2009, while AME_N_ values were estimated using the World’s Poultry Science Association, (1984) formula for compound diets (Table 3).

**Table 3:** Content of nutrients (analyzed^1^; g/kg of original substance) and energy (calculated^2^; MJ AME_N_/kg of original substance) of complete feed mixtures used in rearing (phase 1 and 2) and fattening (phases 3 and 4).

	Phase 1			Phase 2		Phase 3	Phase 4
Item	80 %^3^	80 %^4^	70 %	90 %	80 %	90 %	90 %
Dry matter	911	915	908	914	909	910	899
Crude ash	87	90	83	79	71	66	55
Crude protein	277	280	249	268	241	227	181
Lysine	14.3	14.4	12.3	12.4	10.9	10.8	8.1
Methionine	5.1	5.2	4.5	4.7	3.9	3.9	3.2
Cysteine	4.5	4.8	4.1	4.3	3.8	3.9	3.1
Threonine	10.5	10.8	9.3	9.8	8.5	8.1	6.6
Tyrosine	8.7	8.4	7.5	7.1	6.3	7.0	4.8
Phenylalanine	14.1	13.2	11.6	11.9	10.4	9.4	8.0
Ether extract	76	87	68	79	72	73	65
Crude fibre	52	57	53	72	67	69	65
Saccharose	50	60	48	65	58	61	55
Starch	232	186	278	230	300	310	389
AME_N_	11.4	11.2	11.5	11.5	12.0	12.0	12.2
Lysine/AME_N_^5^	1.25	1.29	1.07	1.08	0.91	0.90	0.66
Methionine/AME_N_^5^	0.45	0.46	0.39	0.41	0.33	0.33	0.26

1 Nutrient contents were determined by wet-chemical analysis according to European Commission (2009) Regulation No. 152/2009.

2 Calculated according to WPSA (1984).

3 Essential amino acid levels (focus: lysine and methionine) used in the experimental diets compared to Aviagen (2015) recommendations for a low energy density feeding program, based on the ratio of lysine and methionine to MJ AME_N_.

4 without pea protein concentrate.

5 in g/MJ.

### Data collection

Individual BW were recorded on days 28, 56, 84, and 112 with a platform scale (RHEWA 83 Sigma, Rhewa-Waagenfabrik, August Freudewald GmbH & Co. KG, Germany). Every two weeks total feed intake was recorded (Defender™ 5000-D52, OHAUS Europe GmbH, Switzerland) in each pen to determine the average feed intake (**FI**) and feed conversion ratio (**FCR**). Data were adjusted for mortality. The animals were examined daily, and any losses were recorded.

Cumulative dietary AA intake of methionine and cysteine, essential for feather keratin synthesis (Deschutter and Leeson, 1986), and of tyrosine and phenylalanine, necessary for melanin-based pigmentation (Gudowska et al., 2022), were calculated. The formula used for the average cumulative AA intake in g was as follows:

$$\text{Cumulative}\;\text{AA}\;\text{intake}\;=\sum_{i=n}^{n}\left(\text{average}\;FI_{i}\times \;\text{AA}\;\text{conten}t_{i}\right)$$

where average FI*_i_* is the average feed intake of phase*_i_*, AA content*_i_* is the analyzed AA content (methionine, cysteine, tyrosine, phenylalanine) of the complete feed mixtures per corresponding phase *i, n* is the total number of phases, with the summation considering the cumulative intake until phase *n*.

Phase-specific AA intake was calculated for each F phase separately using the same formula but without summation across phases.

### Integument scoring

To characterize the condition of the animals and indirectly determine the occurrence of feather pecking and injurious pecking, integument scoring was conducted for all animals at the end of each feeding phase (week 4, 8, 12, and 16). The same observer performed all integument assessments to ensure consistency. Intra-observer reliability was evaluated at three time points during the study (weeks 4, 8, and 12), each with a sample of 50 animals.

**Fig. 1:**
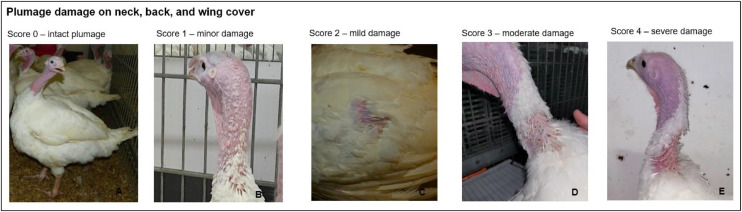
The scoring system for the visual assessment for plumage damage on neck, back, and wing cover. A 5-point scoring system was used to assess plumage damage to the neck, back and wing cover: score 0 (A complete, close-fitting plumage), score 1 (B – individual feathers missing or damaged), score 2 (C – featherless area(s) ≤ 2 cm), score 3 (D – featherless area(s) > 2 to ≤ 8 cm) and score 4 (E – featherless area(s) > 8 cm).

**Fig. 2:**
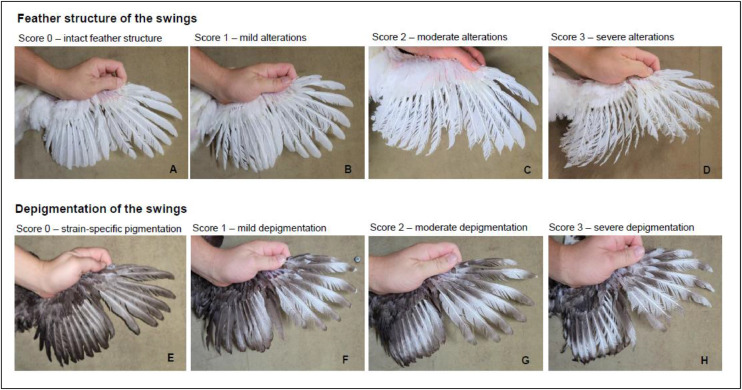
The scoring system for the visual assessment for feather structure and depigmentation of wing. A 4-point scoring system was used in each case, with the definition of scores based on the relative proportion of altered feather area. For feather structure of wings, the following scores were assigned: score 0 (A – ≤ 10 % of the feather area with not closed barbs), score 1 (B – > 10 % to ≤ 40 % of the feather area with not closed barbs), score 2 (C – > 40 % to ≤ 70 % of the feather area with not closed barbs), and score 3 (D – > 70 % of the feather area with not closed barbs). For depigmentation of wings, the following scores were assigned: score 0 (E – ≤ 10 % of the feather area with color lightening), score 1 (F – > 10 % to ≤ 40 % of the feather area with color lightening), score 2 (G – > 40 % to ≤ 70 % of the feather area with color lightening), and score 3 (H – > 70 % of the feather area with color lightening).

Plumage damage on the neck, back (including the sides of the body), wing cover, and wings (primaries and secondaries) was assessed using a 5-point scale ranging from 0 (intact) to 4 (severe damage; Fig. 1), as outlined by Schulze-Bisping (2015). Pecking injuries were categorized into regions including the neck, head, snood, and caruncle, with a scoring scale from 0 (intact skin) to 3 (severe damage), according to Schulze-Bisping (2015). For wing feather structure and depigmentation, a separate 4-point scale was developed due to the absence of an established scoring system for these characteristics (Fig. 2). This 4-level scale for wing feather structure ranged from Score 0 (no alterations) to Score 3 (severe alterations). Depigmentation, characterized by the lightening of colored feathers, was only assessed in Auburn turkeys, as depigmentation was not observable in the white-feathered B.U.T. 6 turkeys. In addition to the individual scores for specific body regions, a total plumage score was calculated by summing the individual scores of neck, back (including sides of the body), and wing cover according to Schreiter et al., (2020). A wing total score was calculated by adding the individual scores for wing plumage damage, feather structure, and depigmentation. In the case of injuries, the neck, head, snood, and caruncle were summarized by adding the individual scores to a total score, as were the individual scores for the back and wings.

### Statistical analyses

***Growth performance.*** Data collection was performed using Microsoft Excel® 2016 (Microsoft Corporation, Redmond, USA). After testing for normal distribution and homogeneity of variances, statistical analyses were conducted using two-way ANOVA with the GLM procedure in SAS 9.4 for Windows (SAS Institute Inc., Cary, NC, USA). The experimental unit for the analysis was the pen (group of animals, *n* = 4 pens as replicates). The main effects of G and F strategies on growth performance and AA intake (methionine, cysteine, tyrosine, phenylalanine) from concentrate were examined, including the effect of their interaction (***G* × *F***). The following statistical model was used:

y_ij_ = μ + G_i_ + F_j_ + G_i_ × F_j_ + e_ij_

where y_ij_ is the dependent trait, μ is the overall mean, G_i_ is the fixed effect of the i^th^ G (i = 1, 2), F_j_ is the fixed effect of the j^th^ F group (*j* = 1, 2, 3), G_i_ × F_j_ is the fixed interaction effect of the i^th^ G and j^th^ F group and e_ij_ is the residual error. Tukey's multiple comparison test was used to determine significant differences between means. Results are expressed as least square means ± pooled standard errors (**SE**). Differences were considered statistically significant at *P* ≤ 0.05.

Additionally, LS-means from the G × F analysis were used to calculate the percentage deviation in the intake of methionine, cysteine, tyrosine, and phenylalanine of both G in F2 and F3, relative to the control group F1. The control group F1 served as the reference point, corresponding to a 0 % deviation. For the calculation, methionine and cysteine, as well as tyrosine and phenylalanine, were each considered as pairs. The LS-means of methionine and cysteine were averaged to obtain the Met + Cys value, and likewise, tyrosine and phenylalanine were averaged to obtain the Tyr + Phe value. The percentage deviation for F2 and F3 was then calculated according to the following general formula

$$\;\text{Percentage}\;\text{deviation}\;\text{for}\;\text{AA}\;\text{intake}=\left(\frac{\text{AA}\;\text{intake}\;\left(F2,\;F3\right)}{\text{AA}\;\text{intake}\;\left(F1\right)}-1\right)\times 100$$

where AA intake (F2, F3) and AA intake (F1)​ represent the respective averaged intakes of Met + Cys or Tyr + Phe for the groups F2 or F3 and the control group F1.

***Integument scoring.*** Microsoft Excel® 2013 (Microsoft Corporation, Redmond, USA) was used for data collection and processing. Statistical analyses were performed using SAS 9.4 for Windows (SAS Institute Inc., Cary, NC, USA) and IBM SPSS Statistics 23 (SPSS Inc., Chicago, USA).

Concordance analysis was conducted to assess intra-observer reliability using prevalence-adjusted and bias-adjusted kappa (**PABAK**; Gunnarsson et al., 2000), with values interpreted as follows: < 0.20 (insufficient), 0.21–0.40 (sufficient), 0.41–0.60 (moderate), 0.61–0.80 (good), and > 0.80 (very good) (Landis and Koch, 1977; Kwiecien et al., 2011).

Binary logistic regression (**BLR**) models were applied to integument traits (Baltes-Götz, 2000). Multiple logistic regression models were applied instead of ordinal models due to the limited number of observations for certain values and the absence of the proportional odds assumption. To enhance statistical power and better identify key determinants, the integument characteristics were dichotomized, as only a few animals exhibited severe alterations. Consequently, ordinal scores were transformed into nominal categories, with a score of 0 indicating no alteration and scores ≥1 indicating any degree of alteration. Risk factors such as age and G (Haug et al., 2023), F, and *G* × *F* interactions were included in the models. A backward selection approach (*P* < 0.10) was used to retain independent variables an interactions while minimizing type II errors and maintaining a 5 % type I error threshold (Hosmer and Lemeshow, 2000). The depigmentation trait was assessed only in Auburn turkeys, omitting G and interaction effects in the BLR model. Multicollinearity was checked by calculating the Pearson correlation coefficients and using collinearity diagnostics with the variance inflation factor (Menard, 1995; Field, 2013), and no multicollinearity was found. Nagelkerke’s R² values were used to assess the proportion of variance explained by the model. Values ≥ 0.50 were considered "very good," and those between 0.40 and 0.50 were considered "good" (Backhaus et al., 2016).

Spearman's rank correlation (Hauke and Kossowski, 2011) was used to analyze correlations between wing features (plumage damage, feather structure, depigmentation), with interpretation following Hinkle et al. (2003).

The flocks were divided into two wing status groups to examine correlations between wing condition (**WC**) and BW. For this classification, each *G* × *F* was categorized based on the median wing total score at week 4. Animals with a score below the median (no/mild wing alterations = WC+) were compared with those above the median (pronounced wing alterations = WC-). ANOVA linear models were used, with WC as a between-subject effect and age as a within-subject effect, to compare changes in BW during the fattening period between animals with different wing condition statuses in the juvenile age (Rasch et al., 2010).

A Cox regression model analyzed survival time regarding animal losses, with animal deaths or exclusions defined as events, and data from slaughtered animals censored (Zwiener et al., 2011). Differences were considered statistically significant for *P* ≤ 0.05.

## Results

The initial BW was 60.3 g for Auburn and 58.5 g for B.U.T. 6 (± 0.02), with no difference observed among F groups (*P* = 0.611), but between G (*P* < 0.001). Three animals were lost during the study, resulting in mortality rates of 0.9 % for B.U.T. 6, 1.9 % for Auburn (*P* = 0.578), 1.4 % for F1, 2.8 % for F2 and 0.0 % for F3 (*P* = 0.849). No deaths were attributed to injury pecking.

### Growth performance

***Feed intake and body weight.*** Interaction effects between G and F were observed for FI and BW (Table 4). In B.U.T. 6, F3 birds consistently exhibited the lowest FI from P1 to P3 and total (*P* ≤ 0.044), and the lowest BW from day 28 to day 84 (*P* < 0.001). No differences were observed between the B.U.T. 6 F1 and F2 groups for FI and BW (*P* ≥ 0.076), except on day 56, when F1 had a higher BW compared to F2 (*P* = 0.001). In contrast, Auburn F3 birds had similar FI to F1 and F2 during P2, P3 and total (*P* ≥ 0.195), but had lower FI during P1 compared to F2 (*P* = 0.025). Despite these G-related differences in FI, Auburn F3 birds had the lowest BW from day 28 to day 84 (*P* ≤ 0.021), reflecting the response observed in B.U.T. 6 F3 birds. However, across G, slow-growing Auburn turkeys partially matched the FI and BW of fast-growing B.U.T. 6 during rearing, particularly B.U.T. 6 F3. In P1, no differences were observed in FI between B.U.T. 6 F3 and Auburn F1 and F3 (*P* ≥ 0.699), and in P2 between B.U.T. 6 F3 and all Auburn groups (*P* ≥ 0.088). BW of B.U.T. 6 F3 showed no differences compared to Auburn F1 and F2 on day 28 (*P* ≥ 0.079) and to Auburn F1 on day 56 (*P* = 0.121).

**Table 4:** Effect of energy- and essential amino acid reduced feeding on growth performance of slow-growing Auburn and fast-growing B.U.T. 6 female turkey strains (Least square means ± pooled standard error (SE)).

Trait	Feed intake (kg/animal)					Body weight (kg/animal)				Feed conversion ratio (kg feed/kg weight gain)				
	P1^1^	P2	P3	P4	P1-P4	d 28	d 56	d 84	d 112	P1	P2	P3	P4	P1-P4
Genotype (G)														
Auburn	1.00	3.72	7.14	9.62^B^	21.5	0.70	2.81	5.59	7.97^B^	1.56^A^	1.77^A^	2.56^A^	4.05^A^	2.71
B.U.T. 6	1.13	4.59	9.66	14.6^A^	30.0	0.84	3.69	7.89	12.1^A^	1.45^B^	1.61^B^	2.30^B^	3.49^B^	2.49
SE	0.019	0.062	0.086	0.144	0.249	0.009	0.027	0.043	0.099	0.029	0.025	0.021	0.056	0.017
Feeding (F)^2^														
F1	1.09	4.53	8.44	12.3	26.3	0.81	3.56	6.97	10.1^AB^	1.45	1.66	2.50^A^	3.96^A^	2.63
F2	1.19	4.12	8.61	12.2	26.1	0.85	3.31	6.92	10.3^A^	1.52	1.68	2.41^AB^	3.76^AB^	2.59
F3	0.92	3.81	8.15	11.8	24.7	0.65	2.87	6.32	9.72^B^	1.55	1.74	2.39^B^	3.58^B^	2.59
SE	0.024	0.076	0.105	0.176	0.305	0.012	0.033	0.052	0.121	0.036	0.031	0.025	0.068	0.021
*G* × *F*														
Auburn F1	0.98^cd^	3.96^b^	7.00^c^	9.63	21.6^c^	0.74^b^	3.03^cd^	5.71^c^	8.06	1.45	1.73	2.61	4.10	2.70^ab^
F2	1.09^bc^	3.60^b^	7.31^c^	9.73	21.7^c^	0.76^b^	2.86^d^	5.73^c^	8.08	1.56	1.71	2.55	4.14	2.71^a^
F3	0.92^d^	3.59^b^	7.11^c^	9.52	21.1^c^	0.61^c^	2.53^e^	5.34^d^	7.78	1.66	1.88	2.53	3.90	2.74^a^
B.U.T. 6 F1	1.20^ab^	5.10^a^	9.87^a^	14.9	31.1^a^	0.89^a^	4.09^a^	8.24^a^	12.2	1.44	1.59	2.38	3.81	2.57^bc^
F2	1.28^a^	4.65^a^	9.91^a^	14.7	30.5^a^	0.93^a^	3.76^b^	8.12^a^	12.5	1.47	1.65	2.27	3.39	2.46^c^
F3	0.92^d^	4.03^b^	9.19^b^	14.1	28.3^b^	0.70^b^	3.21^c^	7.31^b^	11.7	1.44	1.60	2.24	3.26	2.44^c^
SE	0.034	0.107	0.149	0.249	0.431	0.015	0.047	0.074	0.171	0.051	0.043	0.036	0.097	0.029
*P*-value														
G	<0.001	<0.001	<0.001	<0.001	<0.001	<0.001	<0.001	<0.001	<0.001	0.017	<0.001	<0.001	<0.001	<0.001
F	<0.001	<0.001	0.021	0.177	0.003	<0.001	<0.001	<0.001	0.013	0.147	0.190	0.018	0.004	0.241
GxF	0.008	0.008	0.046	0.407	0.035	0.014	0.004	0.004	0.375	0.143	0.066	0.728	0.071	0.027

1 Rearing: phase (P)1 – P2; fattening P3-P4, total: P1-P4; 4 weeks with 28 days per phase.

2 Feeding group see Table 1 ^a-d^ For significant (*P* ≤ 0.05) interactions between main effects: labeled means in a column lacking a common lowercase letter differ significantly (*P* ≤ 0.05) ^A-B^ For cases without a significant interaction (*P* > 0.05), but with significant main effects: labeled means in a column lacking a common capital letter differ significantly within the main effects genotype or feeding (*P* ≤ 0.05).

No interaction effects were observed for FI in P4 or final BW on day 112. However, G affected both FI in P4 and final BW, with B.U.T. 6 showing higher FI and final BW (*P* < 0.001). Feeding strategies had no effect on FI in P4 (*P* ≥ 0.190), but differences in final BW were found, with F3 having lower BW than F2 animals (*P* = 0.013).

***Feed conversion ratio.*** An interaction effect between G and F was observed for total FCR, with higher values in Auburn than in B.U.T. 6 (*P* < 0.001*)*, except for the F1 group where no strain difference existed (*P* = 0.056). Auburn turkeys showed a higher FCR than B.U.T. 6 turkeys from P1 to P4 (*P* ≤ 0.017; Table 4). Feeding affected FCR during P3 and P4, with F3 resulting in lower FCR than F1 (*P* ≤ 0.019).

### Amino acid intake

For all studied AA (methionine, cysteine, tyrosine, and phenylalanine), interaction effects between G and F were observed in each observation period (Table 5).

**Table 5:** Average cumulated methionine, cysteine, tyrosine and phenylalanine intake in g from concentrate feed intake of slow-growing Auburn and fast-growing B.U.T. 6 female turkey strains^1^ (Least square means ± pooled standard error (SE)).

Trait	Methionine				Cysteine				Tyrosine				Phenylalanine			
	P1	P1-P2	P1-P3	P1-P4	P1	P1-P2	P1-P3	P1-P4	P1	P1-P2	P1-P3	P1-P4	P1	P1-P2	P1-P3	P1-P4
Genotype (G)																
Auburn	4.94	20.5	48.3	79.1	4.47	19.3	47.1	76.9	8.20	32.7	82.7	129	13.0	53.6	121	198
B.U.T. 6	5.64	24.9	62.6	109	5.10	23.4	61.1	106	9.36	39.6	107	177	14.8	65.1	156	273
SE	0.092	0.282	0.570	0.905	0.083	0.267	0.554	0.879	0.152	0.449	0.971	1.46	0.238	0.740	1.14	2.27
Feeding (F)^2^																
F1	5.56	26.8	59.8	99.0	4.91	24.4	57.3	95.4	9.49	41.6	101	160	15.4	69.3	149	247
F2	6.17	22.2	55.8	94.9	5.69	21.4	55.0	92.8	9.96	35.9	96.2	155	15.7	58.6	140	237
F3	4.13	19.0	50.8	88.6	3.76	18.2	50.0	86.7	6.88	30.9	87.9	145	10.6	50.2	127	222
SE	0.112	0.345	0.699	1.11	0.102	0.327	0.679	1.08	0.187	0.550	1.19	1.79	0.292	0.906	1.75	2.77
*G* × *F*																
Auburn F1	5.02^c^	23.6^b^	51.0^d^	81.8^c^	4.43^c^	21.5^c^	48.8^c^	78.6^c^	8.56^b^	36.7^b^	85.7^c^	132^c^	13.9^b^	61.0^b^	127^d^	204^c^
F2	5.66^bc^	19.7^c^	48.2^de^	79.3^c^	5.22^b^	18.9^d^	47.4^c^	77.6^c^	9.14^b^	31.8^c^	83.0^c^	130^c^	14.4^b^	51.8^c^	121^de^	198^c^
F3	4.15^d^	18.1^c^	45.9^e^	76.3^c^	3.77^d^	17.4^d^	45.2^c^	74.7^c^	6.90^c^	29.5^c^	79.3^c^	125^c^	10.7^c^	48.0^c^	115^e^	191^c^
B.U.T. 6 F1	6.11^ab^	30.1^a^	68.6^a^	116^a^	5.39^b^	27.3^a^	65.8^a^	112^a^	10.4^a^	46.6^a^	116^a^	187^a^	16.9^a^	77.5^a^	170^a^	290^a^
F2	6.68^a^	24.8^b^	63.5^b^	110^a^	6.16^a^	23.8^b^	62.5^a^	108^a^	10.8^a^	40.1^b^	110^a^	180^a^	17.0^a^	65.3^b^	159^b^	276^a^
F3	4.12^d^	19.8^c^	55.7^c^	101^b^	3.75^d^	19.1^d^	54.9^b^	98.7^b^	6.86^c^	32.2^c^	96.5^b^	164^b^	10.6^c^	52.5^c^	139^c^	252^b^
SE	0.159	0.489	0.988	1.57	0.144	0.462	0.960	1.52	0.264	0.778	1.68	2.53	0.413	1.28	2.47	3.93
*P*-value																
G	<0.001	<0.001	<0.001	<0.001	<0.001	<0.001	<0.001	<0.001	<0.001	<0.001	<0.001	<0.001	<0.001	<0.001	<0.001	<0.001
F	<0.001	<0.001	<0.001	<0.001	<0.001	<0.001	<0.001	<0.001	<0.001	<0.001	<0.001	<0.001	<0.001	<0.001	<0.001	<0.001
GxF	0.004	<0.001	0.003	0.016	0.004	0.001	0.004	0.019	0.004	0.001	0.004	0.016	0.003	0.001	0.003	0.016

1 rearing: phase (P)1 – P2; fattening P3-P4, total: P1-P4; 4 weeks with 28 days per phase.

2 feeding group see Table 1 ^a-e^ for significant *(P* ≤ 0.05) interactions between main effects: labeled means in a column lacking a common lowercase letter differ significantly *(P* ≤ 0.05).

***Cumulative intake during rearing.*** The lowest levels for methionine, cysteine, tyrosine, and phenylalanine intake during P1 were observed in group F3 (*P* ≤ 0.046), with Auburn F3 and B.U.T. 6 F3 showing no difference in intake (*P* = 1.00). For cysteine intake in P1, all F groups within each G differed (F2 > F1 > F3; *P* ≤ 0.046). Throughout the rearing period (P1-P2), B.U.T. 6 F3 animals had the lowest cumulative intake for all studied AA within the G (*P* < 0.001). In contrast, no differences were observed between Auburn F2 and F3 (*P* ≥ 0.251), with B.U.T. 6 F3 having the same intake (*P* ≥ 0.173).

***Cumulative intake including fattening.*** B.U.T. 6 had higher intake for all studied AA compared to Auburn in both P1-P3 and P1-P4 (*P* ≤ 0.034). For cumulative methionine and phenylalanine intake from P1-P3, all F groups within B.U.T. 6 differed (F1 > F2 > F3; *P* ≤ 0.033). In contrast, Auburn F1 had a higher intake than F3 (*P* ≤ 0.020), while F2 did not differ from either (*P* ≥ 0.387). For the cumulative intake of cysteine and tyrosine from P1-P3 and P1-P4, as well as methionine and phenylalanine from P1-P4, no differences were observed among the Auburn F groups (*P* ≥ 0.133). In contrast, within B.U.T. 6, F1 and F2 continued to showed higher intake levels compared to F3 (*P* ≤ 0.005).

**Fig. 3:**
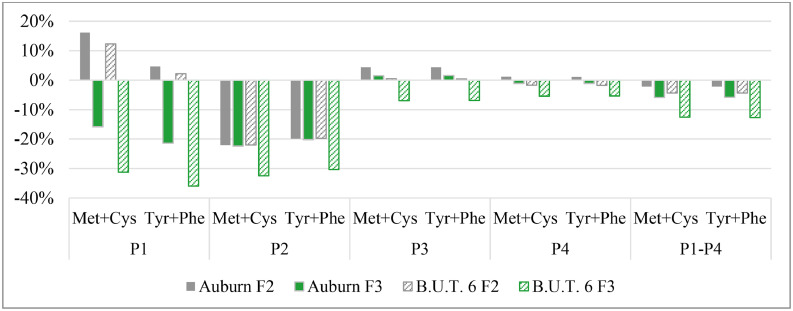
Percentage deviation in phase-specific (P1, P2, P3, P4, 4 weeks per phase) and cumulative (P1-P4) Methionine + Cysteine (Met + Cys) and Tyrosine + Phenylalanine (Tyr + Phe) intake of female Auburn and B.U.T.6 turkeys in feeding groups F2 and F3, relative to the control group F1 (0 % line = reference; F1-F3, see Table 1).

A similar trend was observed for the percentage deviation in phase-specific and cumulative total (P1-P4) intake of Met + Cys and Tyr + Phe in Auburn and B.U.T.6 turkeys from F groups F2 and F3, relative to the control group F1 (Fig. 3).

### Injuries and plumage traits

PABAK values of 0.91 for plumage damage of neck, back, and wing cover, 0.93 for plumage damage of wings, 0.87 for feather structure of wings, 0.82 for depigmentation of wings, 0.96 for injuries of neck, head, snood, and caruncle and 0.94 for injuries of back and wings indicated very good intra-observer reliabilities.

The BLR models analyzed the effects of G, F group, and age on plumage damage (Table 6), wing feather condition (feather structure and depigmentation, Table 7) and pecking injuries (Table 8).

**Table 6:** Effects of genotype, feeding and age on plumage damage in various body regions (logistic regression analysis).

Trait	Nagelkerke R^2^	Score 1 (%)	Coefficients (SE)	Odds ratio (95 % CI)	individual *P*-value	overall *P*-value
Plumage damage^1^ neck/back/wing cover	0.308					
Genotype						
B.U.T. 6		17.1	Reference	Baseline		
Auburn		6.5	−1.18 (0.25)	0.31 (0.19-0.49)		<0.001
Feeding^2^						
F1		7.3	Reference	Baseline		
F2		9.2	0.25 (0.32)	1.29 (0.69-2.40)	0.425	<0.001
F3		18.7	1.19 (0.29)	3.30 (1.86-5.87)	<0.001	
Age						
week 4		0.0	Reference	Baseline		
week 8		0.0	0.00 (0.05)	0.99 (0.31-3.18)	0.998	
week 12		20.9	2.32 (0.45)	10.24 (4.19-25.04)	<0.001	<0.001
week 16		31.0	2.93 (0.46)	18.78 (7.62-46.30)	<0.001	
Intercept			−3.72 (0.48)			
Plumage damage^1^ wings	0.344					
Genotype						
B.U.T. 6		53.5	Reference	Baseline		
Auburn		42.4	−0.58 (0.16)	0.56 (0.40-0.77)		<0.001
Feeding^2^						
F1		37.0	Reference	Baseline		
F2		46.9	0.53 (0.20)	1.70 (1.14-2.52)	0.008	<0.001
F3		59.3	1.19 (0.21)	3.29 (2.19-4.94)	<0.001	
Age						
week 4		43.1	Reference	Baseline		
week 8		73.6	1.39 (0.22)	4.04 (2.64-6.20)	<0.001	
week 12		56.9	0.60 (0.20)	1.82 (1.22-2.73)	<0.001	<0.001
week 16		7.6	−2.33 (0.34)	0.09 (0.05-0.19)	<0.001	
Intercept			−0.58 (0.20)			

1 The genotype x feeding interaction was excluded from the model during variable selection.

2 feeding group see Table 1 SE – standard error, CI – confidence interval.

**Table 7:** Effects of genotype, feeding, age and the interaction between genotype and feeding on feather structure and depigmentation of wings (logistic regression analysis).

Trait	Nagelkerke R^2^	Score 1 (%)	Coefficients (SE)	Odds ratio (95 % CI)	individual *P*-value	overall *P*-value
Feather structure of wings	0.650					
Genotype						
B.U.T. 6		46.0	Reference	Baseline		
Auburn		33.4	−1.81 (0.39)	0.16 (0.07-0.35)		<0.001
Feeding^1^						
F1		29.8	Reference	Baseline		
F2		36.6	0.24 (0.37)	1.28 (0.61-2.68)	0.509	<0.001
F3		52.2	1.47 (0.39)	4.36 (2.01-9.44)	<0.001	
Age						
week 4		85.3	Reference	Baseline		
week 8		52.8	−2.02 (0.27)	0.13 (0.07-0.23)	<0.001	
week 12		7.1	−5.11 (0.38)	0.01 (0.01-0.01)	<0.001	<0.001
week 16		4.4	−5.63 (0.47)	0.01 (0.00-0.01)	<0.001	
Genotype × Feeding			0.84 (0.54)	2.32 (0.81-6.65)		0.048
Intercept			−3.72 (0.48)			
Depigmentation of wings^2^	0.631					
Feeding^1^						
F1		10.7	Reference	Baseline		
F2		13.7	0.42 (0.33)	1.53 (0.79-2.97)	0.204	<0.001
F3		27.2	3.14 (0.51)	23.28 (8.42-64.32)	<0.001	
Age						
week 4		33.2	Reference	Baseline		
week 8		29.7	−0.39 (0.31)	0.67 (0.35-1.27)	0.228	
week 12		1.4	−5.49 (0.75)	0.01 (0.00-0.02)	<0.001	<0.001
week 16		0.6	−4.89 (0.69)	0.01 (0.00-0.03)	<0.001	
Intercept			−0.17 (0.28)			

1 feeding group see Table 1.

2 As this trait could only be observed in Auburn turkeys, the independent variables included in the model were only feeding and age SE – standard error, CI – confidence interval.

**Table 8:** Effects of genotype, feeding and age on skin lesions in different body regions (logistic regression analysis).

Trait	Nagelkerke R^2^	Score 1 (%)	Coefficients (SE)	Odds ratio (95 % CI)	individual *P*-value	overall *P*-value
Injuries neck/head/snood/caruncle^1^	0.259					
Genotype						
B.U.T. 6		16.4	Reference	Baseline		
Auburn		4.7	−1.59 (0.29)	0.21 (0.11-0.36)		<0.001
Feeding^2^						
F1		6.9	Reference	Baseline		
F2		7.6	0.13 (0.35)	1.14 (0.56-2.3)	0.710	<0.001
F3		16.8	1.20 (0.31)	3.33 (1.78-6.22)	<0.001	
Age						
week 4		0.0	Reference	Baseline		
week 8		5.7	0.74 (0.51)	2.11 (0.76-5.83)	0.149	
week 12		11.4	1.57 (0.47)	4.81 (1.88-12.26)	0.001	<0.001
week 16		25.9	2.72 (0.46)	15.19 (6.07-38.02)	<0.001	
Intercept			−3.61 (0.48)			
Injuries back/wings^1^	0.201					
Genotype						
B.U.T. 6		9.2	Reference	Baseline		
Auburn		2.2	−1.58 (0.38)	0.20 (0.09-0.43)		<0.001
Feeding^2^						
F1		4.2	Reference	Baseline		
F2		3.1	−0.33 (0.48)	0.71 (0.27-1.84)	0.486	0.002
F3		9.7	0.99 (0.38)	2.71 (1.27-5.77)	0.010	
Age						
week 4		0.0	Reference	Baseline		
week 8		1.4	1.01 (0.72)	1.15 (0.87-1.74)	0.316	
week 12		8.1	1.16 (0.50)	3.19 (1.21-8.42)	0.019	<0.001
week 16		12.0	1.66 (0.49)	5.26 (2.00-13.85)	0.001	
Intercept			−3.39 (0.51)			

1 The genotype x feeding interaction was excluded from the model during variable selection.

2 feeding group see Table 1 SE – standard error, CI – confidence interval.

Except for feather structure, *G* × *F* interactions were excluded during variable selection due to no significant associations. Plumage damage on the neck, back, and wings, along with skin injuries, was more frequent in B.U.T. 6 than in Auburn (*P* < 0.001) and increased with age after the 8th week of life (*P* < 0.001). Feeding group 1 and F2 showed less plumage damage compared to F3 (*P* < 0.001) and fewer skin injuries of neck, head, snood, and caruncle (*P* < 0.001) and of back and wings (*P* = 0.002). The feather structure model had the highest explanatory power (Nagelkerke R² = 0.650), classified as 'very good'. Feather structure showed improvement with age (*P* < 0.001) and was more altered in B.U.T. 6 than Auburn (*P* < 0.001).

**Fig. 4:**
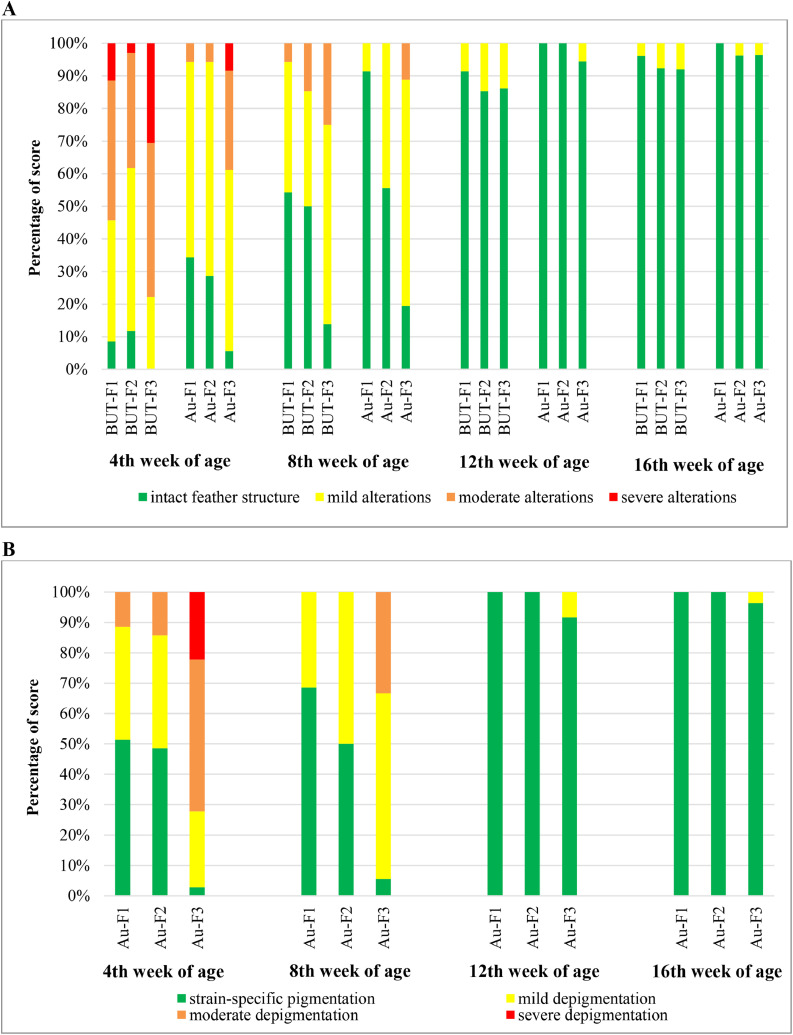
Association of feather structure of wings with genotype (AU: Auburn turkeys; BUT: B.U.T. 6 turkeys), feeding group (F1-F3; see Table 1) and age (A) and depigmentation of wings with feeding and age (B). Scoring scheme is shown in Fig. 2. In logistic regression models, wing depigmentation in Auburn turkeys was significantly associated with feeding and age (each *P* < 0.001). Feather structure of wings was significantly associated with genotype, feeding, and age (each *P* < 0.001), as well as with the genotype × feeding interaction (*P* = 0.048).

The characteristics of feather structure and depigmentation, with strong explanatory power, are visualized in Fig. 4. Feeding group 3 had more feather structure alterations compared to F1 and F2 (*P* < 0.001), with a G × F interaction (*P* = 0.048, Fig. 4A). The increase in altered feather structure compared to F1 was more pronounced in Auburn than in B.U.T. 6 (Score 1 animals at nominal scale: B.U.T. 6-F1 39.7 %, B.U.T. 6-F2 42.2 %, B.U.T. 6-F3 56.1 %, Auburn-F1 19.8 %, Auburn-F2 31.3 %, Auburn-F3 48.5 %). Depigmentation was significantly higher at weeks 4 (33.2 %) and 8 (29.7 %) compared to weeks 12 (1.4 %) and 16 (0.6 %) in Auburn turkeys (Fig. 4B; *P* < 0.001). F1 and F2 showed less depigmentation than F3 (*P* < 0.001).

The correlation analysis for the features of wings revealed a correlation only between feather structure and plumage damage, with moderate correlation at week 4 (rs = 0.595, *P* < 0.001) and low positive correlation at week 8 (rs = 0.471, *P* < 0.001). No correlation was observed at weeks 12 and 16 (rs ≤ 0.170, *P* ≥ 0.159). No correlations between feather loss and depigmentation (rs ≤ 0.157, *P* ≥ 0.159) or between feather structure and depigmentation (rs ≤ 0.272, *P* ≥ 0.243) were observed at any of the observation times.

The linear ANOVA model showed no influence of WC on the development of BW over the fattening period (*P* = 0.985). The BW were as follows: week 4: 0.767 ± 0.133 kg (WC+) vs. 0.776 ± 0.135 kg (WC-); week 8: 3.22 ± 0.612 kg (WC+) vs. 3.26 ± 0.589 kg (WC-); week 12: 6.71 ± 1.29 kg (WC+) vs. 6.74 ± 1.26 kg (WC-); week 16: 10.1 ± 2.24 kg (WC+) vs. 9.98 ± 2.09 kg (WC-).

## Discussion

The objective of the present study was to investigate the effect of reduced dietary AME_N_ and EAA levels on plumage and skin condition as indirect behavioral traits, as well as wing feather structure and plumage depigmentation of two different female turkey strains (Auburn, B.U.T. 6).

***Genotype.*** Genotype significantly influenced growth performance, plumage damage, feather structure, and injuries. B.U.T. 6 turkeys showed higher FI and BW and a lower FCR during fattening compared to Auburn turkeys. However, they also exhibited a higher prevalence of pecking injuries, plumage damage, and altered feather structure, indicating G-specific effects. This aligns with findings by Haug et al. (2023), who reported that male fast-growing B.U.T. 6 turkeys had higher injury and plumage damage rates than slower-growing Auburn turkeys. This is supported by several studies (Busayi et al., 2006; Große Liesner, 2007; Olschewsky et al., 2021) highlighting genetic factors in feather damage and injuries (Busayi et al., 2006). Traditional strains and lighter alternative breeds may benefit from quicker movement, greater submissiveness, more robust feathers and higher feather density, providing higher injury protection compared to genetically selected male line and fast-growing strains, respectively (Busayi et al., 2006; Haug et al., 2023). Other factors, such as light intensity thresholds (Busayi et al., 2006) and earlier maturity in B.U.T. 6 animals (Haug et al., 2023), might also contribute to the expression of antagonistic behaviors. Fewer injuries also mean less bleeding, which could reduce the frequency of pecking (Busayi et al., 2006), whereas the white plumage of B.U.T. 6 turkeys may further affect visibility and attract pecking (Haug et al., 2023). Additionally, Haug et al. (2023) suggested that the larger size and increased BW of B.U.T. 6 turkeys result in more collisions with equipment and longer resting periods, which contribute to greater plumage damage. However, assessing birds at the same BW rather than at the same age could have mitigated these effects, as differences in size and mobility due to genetic background would have been reduced. Only pecking injuries and plumage damage increased with age regardless of G, aligning with research showing that feather damage and injuries become more severe as turkeys age (Leishman et al., 2022; Haug et al., 2023).

***Feeding.*** The greater EAA restriction during rearing (F2, F3) resulted in lower BW compared to higher EAA levels (F1), consistent with findings from other turkey studies (Göppel et al., 2022; Kirn et al., 2024a). The most notable reduction was observed in the F3 group (P1: 70 %; P2: 80 %), where both turkey G exhibited the lowest BWs until day 112. Despite a 30 % EAA reduction during rearing and 10 % during fattening, final BW still met Aviagen (2022a, b) target weights. For F3, B.U.T. 6 even exceeded the target by 0.1 kg and Auburn by 0.5 kg.

The reduction in EAA also affected indirect behavioral traits. Turkeys in F3 showed a significantly higher prevalence of plumage damage, altered feather structure, depigmentation (notably in Auburns), and skin injuries compared to F1 and F2. Animals in F2 had more wing plumage damage than F1, but no other indirect behavioral traits differed. These findings align with previous poultry research, suggesting that low EAA concentrations can affect feather structure and pigmentation (Leeson and Summers, 2008; Kirn et al., 2024b) and contribute to increased injurious and feather pecking behavior (Emous and Krimpen, 2019; Schreiter et al., 2019). This outcome may be attributed to the lower cumulative AA intake in F2 and F3 turkeys during rearing. Emous and Krimpen (2019) highlighted the importance of adequate AA for feather development, particularly in juveniles, as feather growth is energy- and protein-intensive (Leishman et al., 2020). Deficiencies in methionine and cysteine, essential for feather keratin synthesis (Deschutter and Leeson, 1986), can lead to poor feather quality, leading to rough, malformed, and brittle feathers that break easily (Abbott et al., 1962; Emous and Krimpen, 2019). This likely explains the observed correlation between plumage damage and feather structure. Furthermore, nutritional factors can indirectly affect plumage condition by influencing feather-pecking and injury-pecking behavior (Emous and Krimpen, 2019; Schreiter et al., 2019). When dietary requirements are not met, turkeys may redirect their strong beak-related instincts from foraging to harmful pecking behavior (Dalton et al., 2013). In laying hens, inadequate dietary levels of crude protein, lysine, methionine, and crude fiber have been associated with increased frequency of feather and injurious pecking (Schreiter et al., 2019). This injurious pecking can cause feather loss and injuries in specific body areas (Leishman et al., 2022), which may explain the higher prevalence observed in F3 birds. Such behavior is of particular concern due to the potential escalation into cannibalistic pecking. Injurious pecking represents a major welfare and economic concern in turkeys, with cannibalism being a primary cause of mortality (Leishman et al., 2022). However, despite the higher injury prevalence in F3 birds, no differences in mortality were observed between F, and no deaths resulted from injuries. As injurious pecking is a multifactorial behavior influenced by factors such as group size (Leishman et al., 2022), these findings should be interpreted with caution given the small-scale nature of this study.

The darker plumage of Auburn turkeys allowed clear observation of pigmentation changes, revealing significant depigmentation in F3. This can be attributed to the observed lower intake of tyrosine and phenylalanine, which are essential for melanin-based pigmentation (Gudowska et al., 2022). Disrupted melanin synthesis during the molting process prevents tyrosine from forming eumelanin, which leads to a lack of black pigment and results in white plumage (Pomarède, 2000; Poston et al., 2005). These findings are consistent with Gudowska et al., (2022) and Poston et al., (2005), who reported similar feather brightness in house sparrows with reduced tyrosine and phenylalanine intake.

Overall, greater restriction of EAA led to more pronounced alterations in skin and plumage condition, alongside a decrease in BW, with F3 showing the most significant effects. However, no significant correlation was found between mortality and EAA-restricted F, supporting previous poultry studies which indicate that early feed restriction does not lead to increased mortality (Fontana et al., 1992; Shamma et al., 2014). Furthermore, no significant correlation was found between wing condition and BW, suggesting that wing abnormalities do not necessarily affect BW. This is consistent with results in male turkeys, where fault bar traits — visible feather deformities — showed low and insignificant phenotypic correlations with BW (Leishman et al., 2023).

Interestingly, an interaction between G and F was observed, with the increase in altered feather structure in F2 and F3 compared to F1 being more pronounced in Auburn turkeys than in B.U.T. 6. Wylie et al., (2003) demonstrated that in large male turkey lines, feather growth is prioritized over muscle growth even under suboptimal dietary EAA conditions, while in traditional lines, both are similarly affected by protein and EAA restriction. This suggests that genetic differences between Auburn and B.U.T. 6 likely influence how their feather structure responds to nutrient restriction, potentially explaining the more pronounced alterations seen in Auburn turkeys. Additionally, the greater decline in BW observed in F3 B.U.T. 6 turkeys compared to F3 Auburn may be due to B.U.T. 6 prioritizing feather growth over muscle growth under reduced EAA intake. In contrast, Auburn turkeys likely exhibited a more balanced response, with both muscle and feather growth similarly affected by EAA restriction.

The fact that B.U.T. 6 F3 turkeys had a similar EAA intake to that of Auburn F2 and F3 turkeys during rearing, despite their genetic predisposition for faster growth, further supports this finding. However, today’s fast-growing strains may have higher nutritional needs than traditional, slower-growing ones (Chodová et al., 2021; Göppel et al., 2022). These results highlight the importance of considering genetic background when optimizing F strategies, particularly EAA levels, to maintain both performance and welfare across different turkey strains.

Previous studies have shown that an initial reduction in EAA intake during rearing, followed by increased dietary EAA levels during fattening, promotes compensatory feed intake and growth (Göppel et al., 2022; Kirn et al., 2024a). A similar trend was observed in this study, which extends these findings by showing that compensatory feed intake and growth also improve wing condition, including feather structure and pigmentation. Interestingly, despite F2 having a numerically higher AA intake than F1 in P1, no effect on feather structure or pigmentation was observed. However, feather structure alterations and depigmentation decreased with age and increasing dietary EAA levels, as severe alterations were no longer observed by week 8, and moderate changes disappeared by week 12. These improvements may be linked to the increased cumulative AA intake during fattening in both Auburn and B.U.T. 6 turkeys. However, despite similar total cumulative AA intake across all Auburn F groups and B.U.T. 6 F1 and F2 turkeys, tendencies for feather structure alterations were observed during fattening: in B.U.T. 6, F1 was lower than F2 and F3 at both weeks 12 and 16; in Auburn, F1 and F2 were lower than F3 at week 12, and F1 was lower than both F2 and F3 at week 16. For pigmentation, only Auburn F3 showed a higher tendency at weeks 12 and 16. This trend is likely influenced by the molting process, which begins around week 5 with weekly feather replacement, slowing to intervals of 2-3 weeks after the fifth molt (Leishman et al., 2020). During molt, birds must acquire an adequate mix of EAA to ensure normal feather structure and pigmentation. Notably, melanin is incorporated into feathers only during their growth phase, and no further pigment is added once they are fully developed, meaning feather color remains unchanged after molt (Poston et al., 2005). Therefore, molting should be considered when evaluating feather condition to avoid misinterpreting results. Nevertheless, the study demonstrates that a systematic four-week assessment of feather condition may be effective in monitoring AA nutritional status in turkeys under phase-specific F regimes. This approach aligns with previous findings, which suggest that feathers are valuable indicators of nutritional status throughout poultry development (Leishman et al., 2020; Mróz et al., 2022).

## Conclusion

This study underscores the critical role of dietary EAA supply and its impact on the prevalence of injuries and plumage damage in female turkeys under the tested conditions. A reduction of up to 30 % in EAA during rearing led to increased skin injuries, plumage damage, and altered wing feather structure and depigmentation. In contrast, a moderate 20 % reduction in EAA had no effect on injuries or plumage pigmentation while maintaining high growth performance. Notably, the effects of reduced EAA intake on wing feather structure and pigmentation were reversible with increased EAA supply, highlighting the dynamic relationship between AA availability and wing condition. These findings suggest that wing feather structure and pigmentation can serve as valuable indicators of the AA nutritional status in turkeys. The results provide insights for the nutritional management in commercial turkey production, offering a practical approach to monitor dietary AA supply across different G.

## Declaration of Ai and Ai-assisted technologies in the writing process

During the preparation of this work, the authors used DeepL and ChatGPT (OpenAI) in order to assist with translation, grammatical adjustments, and language refinement. After using these tools, the authors reviewed and edited the content as needed and take full responsibility for the content of the publication.

## CRediT authorship contribution statement

**A.I. Kirn:** Conceptualization, Data curation, Formal analysis, Investigation, Methodology, Validation, Visualization, Writing – original draft, Writing – review & editing. **P. Hofmann:** Conceptualization, Methodology, Resources, Writing – review & editing, Investigation. **P.A. Weindl:** Conceptualization, Investigation, Methodology, Resources, Writing – review & editing. **C. Lambertz:** Conceptualization, Methodology, Project administration, Writing – review & editing. **G. Bellof:** Conceptualization, Formal analysis, Funding acquisition, Methodology, Supervision, Validation, Writing – review & editing. **R. Schreiter:** Conceptualization, Data curation, Formal analysis, Investigation, Methodology, Validation, Visualization, Writing – original draft, Writing – review & editing.

## Disclosures

The authors declare that they have no known competing financial interests or personal relationships that could have appeared to influence the work reported in this paper.
